# Metal (Cd, Cr, Ni, Pb) removal from environmentally relevant waters using polyvinylpyrrolidone-coated magnetite nanoparticles†

**DOI:** 10.1039/c9ra10104g

**Published:** 2020-01-20

**Authors:** Jie Hong, Junyu Xie, Seyyedali Mirshahghassemi, Jamie Lead

**Affiliations:** College of Environment, Zhejiang University of Technology Hangzhou Zhejiang 310014 China; Center for Environmental Nanoscience and Risk, Department of Environmental Health Sciences, Arnold School of Public Health, University of South Carolina Columbia SC 29208 USA jlead@mailbox.sc.edu; College of Resources and Environment, Shanxi Agricultural University Taigu Shanxi 030801 China

## Abstract

Water pollution is a major global challenge given the increasing growth in industry and human population, and certain metals can be highly toxic and contribute to this significantly. In this study, polyvinylpyrrolidone-coated magnetic nanoparticles (PVP–Fe_3_O_4_ NPs) were used to remove metals (Cd, Cr, Ni, and Pb) from synthetic soft water and sea water in the presence and absence of fulvic acid. Nanoparticle (NP) suspensions were added to water media at a range of metal concentrations (0.1–100 mg L^−1^). Removal at different time points (1.5, 3, 6, 12, 24 hours) was also evaluated. Results showed that 167 mg L^−1^ PVP–Fe_3_O_4_ NPs could remove nearly 100% of four metals at 0.1 mg L^−1^ and more than 80% at 1 mg L^−1^. The removal decreased as the initial metal concentration increased, although essentially 100% of the Pb was removed under all conditions. The kinetic adsorption fitted well to the pseudo-second-order model and in general, the majority of metal adsorption occurred within the first 1.5 hours. These NPs are a reliable method to remove metals under a wide range of environmentally relevant conditions. Our previous research showed the NPs effectively removed oil from waters, so these NPs offer the possibility of combined *in situ* remediation of oil and metals.

## Abstract 2

PVP–Fe_3_O_4_ NPs synthesized with no organic solvents, low toxicity reactants and low temperature/energy requirements could remove Cd, Cr, Ni, Pb efficiently in the different synthetic water media under environmentally relevant conditions.

## Graphical abstract

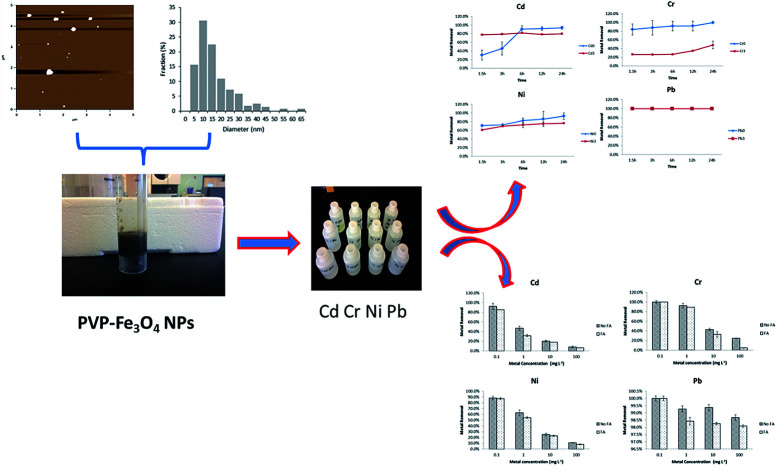

## 1. Introduction

Metals, such as cadmium (Cd), chromium (Cr), nickel (Ni) and lead (Pb) are potentially highly toxic. These metals have a number of adverse outcomes for human health, including kidney failure, softening of bones, prostate cancer, and damage to the liver, children's central nervous system and the reproductive system.^1–4^ These metals are a potential risk, given these hazards and their wide exposure in the environment including their bioaccumulation and bio-magnification.^5,6^ The United States Environmental Protection Agency (USEPA) has set the maximum contaminant level (MCL), which is the highest level of a contaminant that is allowed in drinking water, at 0.005 mg L^−1^ for Cd, 0.1 mg L^−1^ for Cr, 0.1 mg L^−1^ for Ni and 0.015 mg L^−1^ for Pb.^7^ Recently, lead contamination (Pb level was 13–800 times higher than the EPA's MCL) in the City of Flint, MI caused serious environmental health issues.^8^ For soil exposure, our previous work has suggested that MCLs are not sufficiently protective for lead level in soil.^9^ We found a threshold value for low birth weight is about 130.5 mg kg^−1^ Pb in soil, which is lower than the EPA's hazardous value of 400 mg kg^−1^.^10^

To date, many technologies have been used for metal-contaminated sites, such as stabilization, solidification, soil flushing, chemical reduction/oxidation, electrokinetics, low temperature thermal desorption, incineration, excavation/retrieval, disposal and landfill.^11^ However, these technologies are typically expensive and destructive. Phytoremediation may be a better option and less perturbing, but can be time consuming and disposal of the metal-contaminated plant material can be problematic.^12^ All available options therefore have benefits but also limitations and the need for new methods is pressing.

Recently, nanotechnology has been shown to provide a potentially cheap and effective solution for environmental remediation.^13^ Reactive nanomaterials such as nanoscale zeolites, metal oxides, carbon nanotubes, and bimetallic nanoparticles have been used for metal remediation.^14^ Iron oxide nanoparticles are widely used for metal remediation due to their low toxicity and easy separation from water media;^15,16^ in addition, where the NP is composed of magnetite, a facile magnetic separation of NPs, along with associated contaminants, can be performed. However, bare magnetite nanoparticles rapidly aggregate in aqueous systems and are highly susceptible to transformations under many environmental conditions,^17,18^ necessitating the use of appropriate capping agents. For instance, Fe_3_O_4_@SiO_2_ magnetic nanoparticles coated with poly(1-vinylimidazole) oligomer have been used to remove Hg(ii) from water,^19^ while carbon-encapsulated nano-magnets have been used to extract metals even in acidic solutions.^20^ Ferromagnetic carbon-coated Fe nanoparticles have been use to remove nearly 95% Cr(vi) from aqueous solution,^21^ while nanoparticles with a magnetic core and a porous carbon shell could remove metals in acidic suspensions, with high efficiency through electrostatic attraction and adsorption.^22^

Previous work by the authors has shown that polyvinylpyrrolidone coated nanoparticles have great stability under relevant environmental conditions and that PVP is largely non-toxic.^23–25^ A new, facile and cost-effective hydrothermal synthesis technique, using no organic solvents and low temperature/energy requirements and low toxicity reactants was developed to produce PVP-coated Fe_3_O_4_ NPs.^26–28^ It has been shown that these synthesized NPs have a large capacity to remediate oil under environmentally relevant conditions.^26,27^ Here we extend their use to metal remediation (Cd, Cr, Ni, Pb) in the different synthetic water media under various conditions. Metal removal efficiency was evaluated under realistic conditions and the kinetics of adsorption of metals ions was quantified.

## 2. Methodology

### 2.1 Chemicals/materials

Polyvinylpyrrolidone (*M*_w_ 10 kDa), cadmium nitrate (Cd(NO_3_)_2_·4H_2_O, 99%), lead nitrate (Pb(NO_3_)_2_, 99%), nickel nitrate (Ni(NO_3_)_2_·6H_2_O, 99%), potassium dichromate (K_2_CrO_7_, 99%) were purchased from Sigma-Aldrich. FeCl_3_·6H_2_O (>98%) and ammonium hydroxide (NH_4_OH, 25–30%) were purchased from BDH and FeCl_2_·4H_2_O (98%) from Alfa Aesar. All chemicals were used as received without further purification.

### 2.2 Preparation and characterization of PVP–Fe_3_O_4_ NPs

The PVP–Fe_3_O_4_ NPs synthesis used the method published before.^26^ Firstly, 28.8 mM of PVP was added to 6.25 mL ultrapure water (UHP, maximum resistivity 18.2 MΩ cm) while the solution was stirred at 80 ± 5 °C. Subsequently, 160 mM FeCl_2_·4H_2_O and 640 mM FeCl_3_·6H_2_O were added to the solution while the solution was stirred and the temperature was kept constant. Next, 19.2 mM PVP was dissolved in the solution. Finally, 6.25 mL ammonium hydroxide was added into the solution dropwise with vigorous stirring and the solution was mixed for 25 minutes at 90 ± 5 °C and then taken off the heat. After the precipitates reached room temperature, they were washed once with ultrapure water and separated using a 1.5 in. cubic neodymium magnet (Grade N 52, K&J Magnetics Inc.) and redispersed in ultrapure water by sonication. Characterization of the NPs was performed by atomic force microscopy (AFM), Fourier transform infrared spectrometer (FTIR), dynamic light scattering (DLS) and capillary electrophoresis.

### 2.3 Metals adsorption analysis

For all experiments, NP concentrations of 167 mg L^−1^ were used. Suspensions were sonicated for 30 min and shaken (200 rpm) at 25 °C for different time periods. PVP–Fe_3_O_4_ NPs were then separated by a cubic magnet (Grade N 52, K&J magnetic Inc.) until the NPs were completely separated from the aqueous phase. The supernatant was then collected for metal element analysis by inductively coupled plasma optical emission spectrometry (ICP-OES).

Effects of metal concentration, species, water media, and contact time were evaluated. Different concentrations (0.1, 1, 10 and 100 mg L^−1^) of Cd(ii), Cr(vi), Pb(ii), Ni(ii) in aqueous solutions were prepared separately by dissolving their respective nitrate or potassium salt. These concentrations were selected based on a concentration range frequently observed in contaminated waters.^29^ To evaluate the removal efficiency under realistic environmental conditions, two aqueous test media (EPA soft water and marine water) were used, either with or without 0.5 mg L^−1^ of added Suwannee River Fulvic Acid (SRFA). The synthetic soft water and seawater solutions were prepared following the U.S. EPA protocol.^30^ NPs and metals were mixed together for different time periods of 1.5 h, 3 h, 6 h, 12 h, and 24 h, which were used to examine the effects of contact time on metal removal efficiency. In addition, effect of PVP–Fe_3_O_4_ NPs age was also evaluated. Freshly synthesized and three week old PVP–Fe_3_O_4_ NPs were used to remove metals (1 mg L^−1^) from soft water. The three weeks old NPs were sonicated 30 min before addition into metal solutions to help redisperse.

Metal adsorption per unit of adsorbent at time *t* was calculated by eqn (1).^31^1
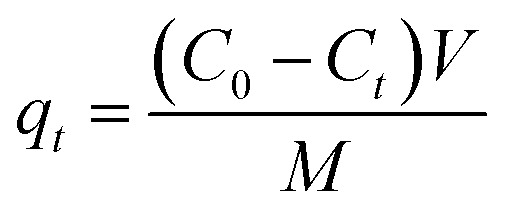
where *C*_0_ (mg L^−1^) is the initial metal ion concentration, *C*_*t*_ (mg L^−1^) is the concentration after adsorption at time *t*. *V* (L) is the solution volume and *M* (g) is the mass of adsorbent.

The removal percentage was calculated by eqn (2):2
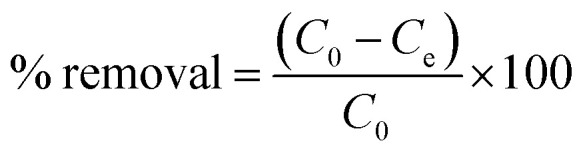
where *C*_0_ and *C*_e_ are the initial and final concentrations of metal ion in the solution. All adsorption experiments were conducted in triplicate and the mean of three values was expressed as the result.

### 2.4 Modeling kinetics of adsorption

In order to investigate the mechanism and rate of the metal adsorption process, a pseudo-first-order equation^32^ was used to fit the data:

3 ln(*q*_e_ − *q*_*t*_) = ln *q*_e_ − *k*_1_*t*

where *q*_e_ and *q*_*t*_ are the amounts of solute adsorbed (mg g^−1^) at steady state and at time *t* (h), respectively, and *k*_1_ (h^−1^) is the rate constant adsorption.

In addition, a pseudo-second-order kinetic model was used, which is given by the following equation:^33^

4 *t*/*q*_*t*_ = 1/*k*_2_*q*_e_^2^ + *t*/*q*_e_ (*k*_0_ = *k*_2_*q*_e_^2^)

where *k*_2_ is the rate constant of the pseudo-second-order model of adsorption. The straight line plots of *t*/*q*_*t*_*versus t* are used to obtain the constants for pseudo-second-order reaction. *k*_0_ is the initial adsorption rate.

### 2.5 Statistical analysis

The reported data in this study are means of three replicates ± standard deviation (SD). One-way ANOVA was used to analyze the experiment variance (SPSS19.0 package, Chicago, IL) followed by the Tukey Test to determine statistical differences between treatments at “*p* < 0.05” level.

## 3. Results and discussion

### 3.1 Characterization

Full information of the PVP–Fe_3_O_4_ characterization was given in the previous study.^26^ In brief, the median particle size and hydrodynamic size is 11.2 nm (interquartile range: 6.3–18.3 nm) and 127.4 ± 4.2 nm as measured by atomic force microscopy (AFM) and dynamic light scattering (DLS), respectively. The Fourier Transform infrared spectrometer (FTIR) result suggests that NPs are coated by PVP and likely through the PVP carbonyl group. In addition, thermogravimetric analysis (TGA) shows that 8.5% of mass of NPs belong to the PVP coating and 91.5% to the iron oxide cores. Based on X-ray diffraction (XRD), magnetite (Fe_3_O_4_) is the dominant phase of NPs.

### 3.2 Metal removal in inorganic solutions

**Fig. 1:**
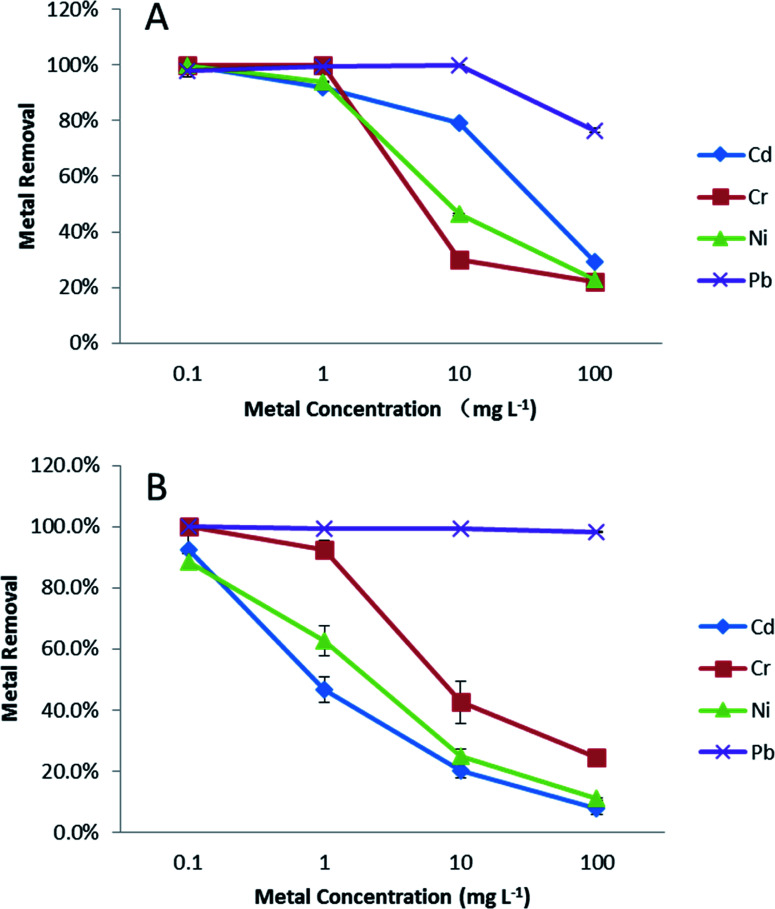
Effects of metal speciation on PVP–Fe_3_O_4_ NPs removal efficiency in different inorganic water media. (A) EPA soft water, (B) EPA sea water. Data are average of three replicates.

The metal removal efficiency using PVP–Fe_3_O_4_ NPs in EPA soft water (A) and EPA sea water (B) is shown in Fig. 1. In Fig. 1A (soft water), it is clear that at lowest metal concentration (0.1 mg L^−1^), removal was nearly complete for all four metals, while this was greater than 90% at 1 mg L^−1^ level of metals. At the highest concentrations (10 and 100 mg L^−1^), only Pb achieved an acceptable removal percentage (≥80%). In Fig. 1B (sea water), the removal percentages were above 90% for all four metal at 0.1 mg L^−1^ level, but at higher level (1 mg L^−1^) only Pb and Cr had more than 90% removal percentage. As with Fig. 1A, all metals when lower than 1 mg L^−1^ were reduced to acceptable concentrations, based on the Criterion Maximum Concentration (CMC) from US EPA. For Cd, Cr, Ni and Pb the CMC values are 18, 16, 470 and 65 μg L^−1^ in freshwater and 33, 1100, 74 and 210 μg L^−1^ in sea water.^34^ At the higher concentrations, only lead was reduced to an CMC ‘acceptable’ level, although addition of further nanoparticles would likely have improved removal. Typical metal concentration in freshwater and seawater are around or lower than CMC value and polluted systems can reach 0.38–12 mg L^−1^.^8,35,36^ Given this condition, PVP–Fe_3_O_4_ NPs could potentially control existing pollution effectively and be used to polish relatively clean waters in a cost effective manner. Addition of more NPs could make them suitable for remediation of more polluted waters, but the cost implications would need to be evaluated on a site-specific basis.

In general, Fig. 1 shows that the removal percentages in soft water (Fig. 1A) were higher than sea water (Fig. 1B). The IEP for Fe_3_O_4_ NPs is at pH 6.5,^37^ although this value will depend on the nature of the NPs and these PVP coated particles show a non-pH dependent slightly positive charge.^26^ The different results for the two water types are most easily explained by charge shielding and neutralization leading to reduced electrostatic attraction between NPs and metals, although partly permeable NPs of this type show much more complex interactions with solutes and solvent.^38^ In the aqueous solution, metal ions can present as stable ions or hydrolyse to form a series of mononuclear and polynuclear hydroxyl as the following reaction^39^M^*n*+^ + *n*H_2_O ↔ M(OH)_*n*_^(*m*−*n*)−^ + *n*H^+^where M stands for metal. Similarly, iron oxide nanoparticle acquire positive or negative charge by protonation/deprotonation in the aqueous solution depends on the solution pH. The process is shown as the following reaction:^40^H_2_O + Fe–O^−^ ↔ Fe–OH ↔ Fe–OH_2_^+^

Therefore, the adsorption of metal ions onto the surface of iron oxide nanoparticles is likely to be an electrostatic attraction between the positive metal ions and negatively charged surface of iron oxide.

It is clear that Pb has a higher adsorption to PVP–Fe_3_O_4_ NPs than other three metals. The binding is affected by differences in the metal's molecular mass, ion charges, ionic radius, hydration energy, and electrostatic and diffusional effects of the polymer coating.^38,41^ The ionic radius of Cr(vi), Ni(ii), Cd(ii) and Pb(ii) are 58, 83, 109, and 133 pm, respectively.^42^ Pb(ii) has the highest ionic radius, which resulted in higher binding power and higher adsorption. Other studies have shown that iron oxide NPs adsorb Pb much more strongly compared to other metals (*e.g.* Cu, Cd) in synthetic and natural water.^43–45^ In soft water (Fig. 1A), at higher concentrations (10 and 100 mg L^−1^), the metal removal followed the trend that Cr < Ni < Cd < Pb, which was same as their ionic radius sequence.

The effects of different metal initial concentrations on removal percentage are shown in Fig S1.† It was shown that, when initial concentration of metal was increased, removal efficiency decreased, as expected of an adsorption reaction. These results are similar as other studies^15,46,47^ and can be explained by the adsorption capacity and the chelating power of the ligand. At low metal concentration, there were more free and stronger binding sites on the surface of NPs, which caused higher metal removal efficiency. The maximum metal binding capacity of NPs limits their effectiveness at high metal concentration.

### 3.3 Effects on metal adsorption of the presence of Suwannee River fulvic acid

**Fig. 2:**
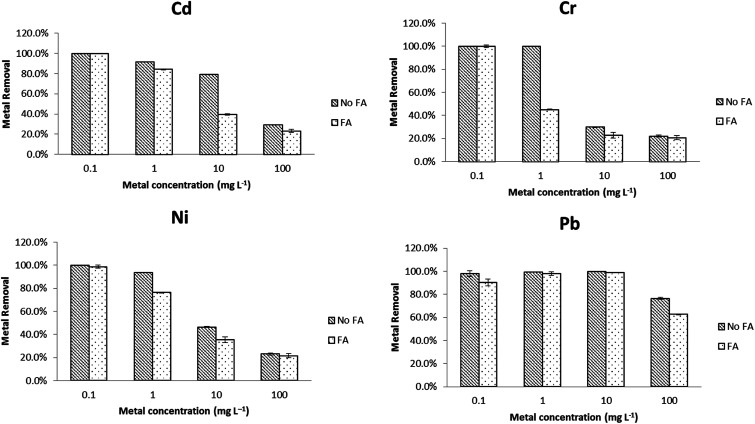
Effects of adding fulvic acid (FA) on metal removal efficiency in soft water.

**Fig. 3:**
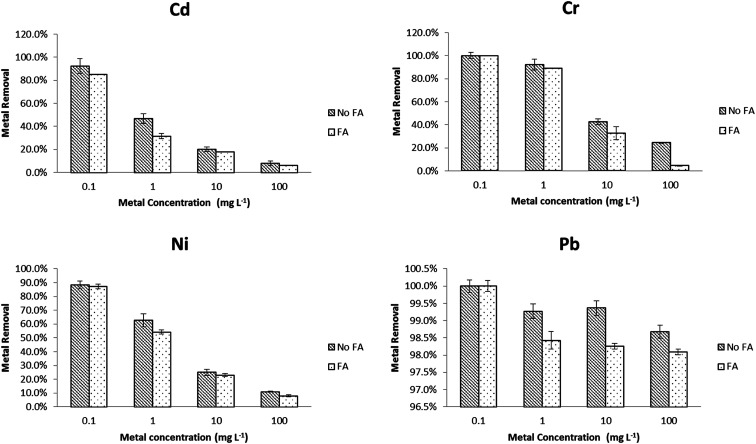
Effects of adding fulvic acid (FA) on metal removal efficiency in sea water.

To better mimic real environmental conditions, metal removal tests in the same EPA water media were performed in the presence of 0.5 mg L^−1^ SRFA and compared with data from Section 1. The effects of adding SRFA to synthetic soft water and sea water are shown in Fig. 2 and 3, respectively. In soft water, there was no significant change (*p* < 0.05) for Pb with the addition of SRFA. For Cd, Cr and Ni, there was no significant change (*p* > 0.05) at 1 mg L^−1^ and 100 mg L^−1^ level. However, the addition of SRFA decreased the removal percentage from 80% to 40% and from 99% to 40% of the 10 mg L^−1^ Cd and 1 mg L^−1^ Cr, respectively. SRFA also significantly decreased Ni removal percentage more than 10% at 1, 10 mg L^−1^. At the lower metal concentrations, there was an excess of free surface sites of PVP–Fe_3_O_4_ NPs for metal binding; at the higher metal concentrations, the free binding sites of the NPs were saturated. Thus, SRFA could not affect metal binding at either very low (1 mg L^−1^) or very high (100 mg L^−1^) metal concentrations, only at the intermediate ratios were the magnetite and SRFA effectively in competition for metal interaction. SRFA had less effect in sea water compared with soft water. As shown in Fig. 3, there was no significant difference for Pb removal percentage after adding SRFA. However, binding significantly decreased 10% of Cd and Ni removal percentage at 1 mg L^−1^, and 20% of Cr removal percentage at 100 mg L^−1^. This simple picture of competition, is complicated by the possibility of SRFA aggregation in sea water^48^ and ternary interactions.^49^

The adsorption capacities of PVP–Fe_3_O_4_ NPs at 10 mg L^−1^ metal level are shown in Table 1. In soft water without SRFA, the highest adsorption capacity of PVP–Fe_3_O_4_ NPs occurred for Pb followed by Cd, Ni and Cr, although Cd and Cr were comparable in the presence of SRFA. In seawater, the sequence was Pb, then Cr, with Ni and Cd comparable. In addition, in the presence of SRFA metal binding was reduced, in all cases and generally to a significant degree (*p* < 0.05). In general PVP–Fe_3_O_4_ had higher adsorption capacities in softer water than sea water, and adding SRFA significantly decreased adsorption under certain conditions. A comparison from the recent literature shows that the magnetite NPs used here in soft water had higher Pb binding capacities: 59.6–61.6 mg g^−1^ than magnetite nanospheres: 18.47 mg g^−1^,^29^ Fe_3_O_4_/SiO_2_ nanocomposites: 17.65 mg g^−1^,^50^ MnO nanocomposites: 21 mg g^−1^,^51^ and magnetic ion-imprinted polymer NPs: 48.1 mg g^−1^.^52^ Also, our NPs had higher adsorption capacities: 17.9–25.5 mg g^−1^ for Cr than other materials, such as magnetite nanospheres: 8.9 mg g^−1^,^29^ amino-modified Fe_3_O_4_ NPs: 11.2 mg g^−1^,^53^ and ceria hollow nanospheres: 15.4 mg g^−1^.^54^ In addition, the adsorption capacities (15.01–29.86 mg g^−1^) for Ni were higher than Fe_3_O_4_ NPs: 11.53 mg g^−1^.^55^ In general, these NPs were highly effective for Pb removal from the aqueous phase and sufficiently so for the other metals tested.

**Table 1:** PVP–Fe_3_O_4_ Adsorption capacity (mg g^−1^) of four metals at 10 mg L^−1^ in different water media. Data are mean value of three replicates ± standard deviation

	Soft water		Sea water	
	Without SRFA	With SRFA	Without SRFA	With SRFA
Cd	43.92 ± 2.56	23.66 ± 2.32	12.08 ± 0.55	11.62 ± 1.03
Cr	17.98 ± 0.86	13.87 ± 0.98	25.52 ± 1.02	22.89 ± 1.78
Ni	29.86 ± 1.45	21.23 ± 0.97	15.01 ± 1.39	13.47 ± 0.65
Pb	61.67 ± 4.57	55.33 ± 3.41	59.62 ± 3.07	20.19 ± 1.49

The reduced metal binding capacity in the presence of SRFA could be explained by the following different mechanisms: (1) SRFA could cause aggregation of PVP–Fe_3_O_4_ NPs, reducing specific surface area and affecting adsorption capacity. Earlier studies showed no aggregation using PVP as a capping agent.^24^ However, at these higher NP concentrations, increased aggregation has been observed.^56^ (2) SRFA might have modified the surface structure of the Fe_3_O_4_ NPs causing changes in crystallinity and degradation of the NPs as we have seen with PVP-coated ceria NPs.^57^ The changed crystal structure could have resulted in reduced magnetic separation. (3) The SRFA could have act as a competitive phase for either PVP–Fe_3_O_4_ NPs or metal ions which could affect adsorption capacity negatively.^58^ Such well known competition (between metal ions and other ions in the sea water) also could explain why the adsorption capacities were lower in sea water than soft water for Cd, Ni and Pb. However, adsorption capacity on Cr is higher in sea water than softer water, possibly related to the dominance of the anion CrO_4_^2−^ (Visual MINTEQ 3.1; Tables S2–S5†).

### 3.4 Kinetic studies

**Fig. 4:**
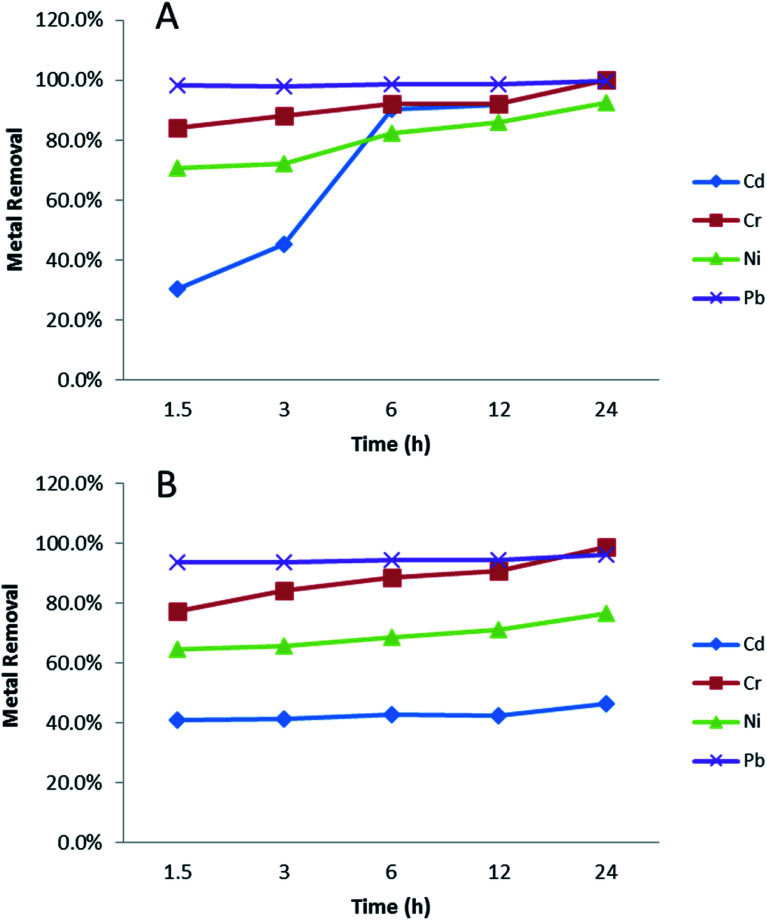
The influence of contact time in metal (1 mg L^−1^) adsorption. (A) EPA soft water, (B) EPA sea water. Data are average of three replicates.

Binding kinetics is important when considering water treatment applications; rapid binding reduces treatment time and cost, while longer contact times may allow greater removal. Fig. 4 shows effects of contact time on metal adsorption over 24 hours, using initial metal concentration at 1 mg L^−1^ in soft water (Fig. 4A) and sea water (Fig. 4B) as examples. In soft water (Fig. 4A), Pb binding was immediate and complete (100% bound at earliest time). For Cr and Ni, ≥75% of all metal was adsorbed within 1.5 hours, with increases over 24 hours, becoming complete after 24 hours (84.0% to 100%, and 70.8% to 92.6%, respectively). Between 1.5 and 24 hours Cd binding significantly increased from 30.3% to 93.5%. In sea water (Fig. 4B), there was no difference for Pb. However, Cr removal percentage significantly increased from 77.2% to 98.8% during 24 h. Cd and Ni had less than 10% removal percentage increase from 1.5 h to 24 h. It is obvious that in sea water, Cd and Ni had lower removal percentage and slower adsorption process compared with soft water. This might be caused by salt in the sea water, which could compete with metal ions binding with NPs.

PVP–Fe_3_O_4_ NPs removed nearly 100% Pb, and most (>60%) of Cr and Ni in the first 1.5 h of mixing. This measured reaction time is shorter than some other studies, such as 2.5 hours for activated carbon^59^ and 4 hours for clay minerals.^60^ However, it was longer than for some other materials, such PMDA/TMSPEDA hybrid polymeric nano-composite^61^ and graphene oxide-based microbots.^62^ Nevertheless, the low cost, simplicity and low environmental footprint of the magnetite NPs^27,28^ may mean they are economically viable.

**Fig. 5:**
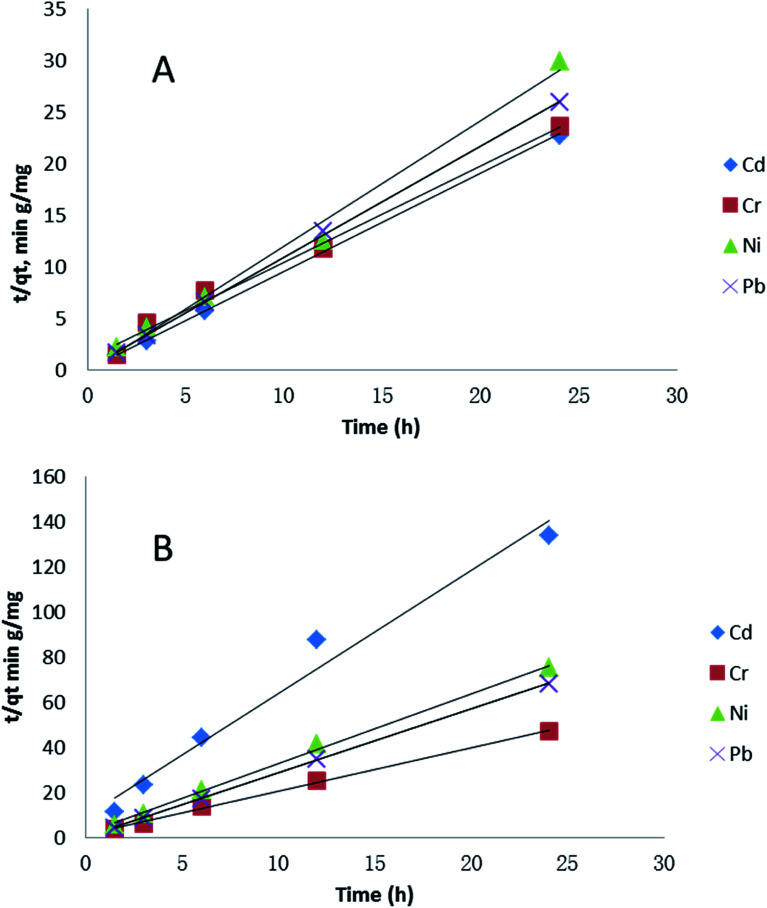
Effect of contact time on pseudo-second-order kinetics of four metals adsorption (1 mg L^−1^ initial metal concentration) in different water media: (A) softer water and (B) sea water.

Adsorption kinetic models allow the estimation of adsorption rate and provide insights into rate expression characteristics of possible reaction mechanisms.^63^Fig. 5 shows the results of a pseudo-second-order adsorption kinetic adsorption model for the four metals in two media, showing similarities due to the fact that most adsorption occurs prior to the 1.5 hour time point. This time point was chosen as the first due to logistical/time constraints in the experiments. Cd adsorption in seawater was the only one which showed a difference, due to the low initial adsorption followed by rapid adsorption between in its major species in soft water was Cd(CO_3_)_2_^2−^ and in sea water were CdCl^+^ and CdCl_2_ (aq) (Table S2†). Cd(CO_3_)_2_^2−^ has negative charge, so it could bind with PVP–Fe_3_O_4_ NPs easily. However, in Fig. 4B there was almost no changes over time for Cd. The possible reason might be that the adsorption occurred at the very beginning and was done before the measurement starts (1.5 h). This assumption was also supported by Fig. 5B, where Cd had a different trend with other three metals. Also, major species of Cr in both water media was CrO_4_^2−^ (Table S3†), which might explain why Cr adsorption had similar trend in both water media. It seems metal speciation could affect adsorption kinetic.

The values of correlation factor *R*^2^, obtained from the plots of pseudo-second-order kinetics shown in Table S1† are greater (*R*^2^ > 0.99) than that of the pseudo-first-order. This suggests that adsorption of Cd, Cr, Ni and Pb onto PVP–Fe_3_O_4_ NPs follows a pseudo-second-order binding. The pseudo second-order adsorption model is based on the assumption that the rate-controlling step of adsorption involved covalent bond through sharing or exchange of electrons between adsorbent and adsorbate,^64^ which means the rate depends on concentrations of PVP–Fe_3_O_4_ NPs and the relevant metal. The pseudo-second-order has also been reported for some heavy metals on many adsorbents such as functionalized magnetic mesoporous silica,^65^ polyethylenimine grafted magnetic porous adsorbent,^66^ monodispersed magnetite NPs.^15^

### 3.5 Different storage period of NPs

**Fig. 6:**
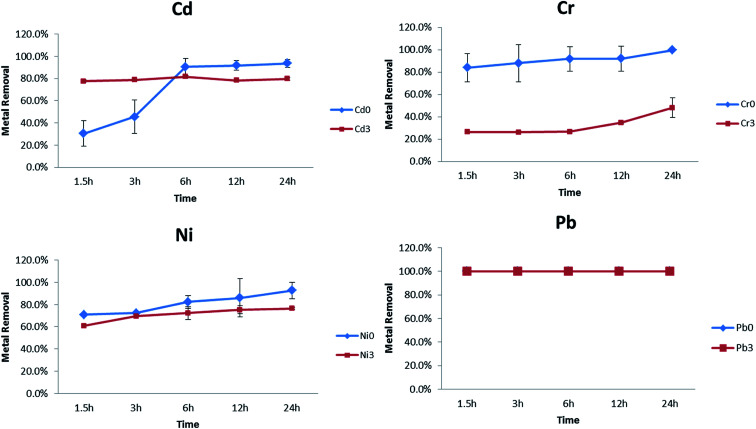
Comparison between fresh and aged (3 weeks old) synthesized nanoparticles' efficiency to remove metals from soft water. Metal concentrations were 1 mg L^−1^.

Practical use of these NPs for treatment, likely requires their production and storage for later use. In order to quantify the effects of ageing, freshly synthesized PVP–Fe_3_O_4_ NPs which had been aged for 3 weeks at room temperature. Results are shown in Fig. 6 for 1 mg L^−1^ in soft water. There was no difference between these two NPs (fresh and stored) in terms of Pb removal efficiency, again showing excellent removal after the storage period. However, there was a large and significant reduction in the NPs ability to remove Cr. The removal percentage is above 80–100% for fresh NPs but only 20–40% for aged ones. In the case of Cd, removal percentages were identical after 6 hours for both the fresh and stored NPs. However, the stored NPs had a reduced capacity at shorter time periods. Possible reason for these differences include agglomeration of NPs leading to reduced specific surface area, or microbial growth leading to several potential alterations. Dilution from stock solutions could also explain the Cd removal before 6 hours, since agglomeration for these NPs is concentration dependent the concentration range.^56^ Dilution into the test solution likely promotes dispersion but with a time delay. In future work, with the exception of Pb, freshly synthesized NPs should be used, or storage conditions investigated further *e.g.* NPs should be diluted prior to use, stored at low temperature.

## 4. Conclusion

In this study, we used PVP–Fe_3_O_4_ NPs for metal remediation in different water media and time, designed to mimic real environmental conditions. Results showed these NPs could reduce metals efficiently in both soft water and seawater. Especially for Pb, the adsorption could be done in less than 1.5 hour and the removal percentage could achieve 100%. The equilibrium data were fitted well by the pseudo-second-order kinetic model. In addition, PVP–Fe_3_O_4_ NPs are synthesized through a facile, environmental friendly and cost-effective hydrothermal technique. In previous study, it has been proved that PVP–Fe_3_O_4_ NPs could remove oil efficiently under different environmental conditions. Therefore, it is expected that PVP–Fe_3_O_4_ NPs have wide applicability in the water remediation. Further investigation is required related to storage conditions in the feasibility of using the NPs in a commercial setting.

## Conflicts of interest

The authors declare no competing financial interests.

## Supplementary Material

RA-010-C9RA10104G-s001
