# Supplementary material for: Metal (Cd, Cr, Ni, Pb) removal from environmentally relevant waters using polyvinylpyrrolidone-coated magnetite nanoparticles
Source: RSC Adv. 2020 Jan 20;10(6):3266–76. doi: 10.1039/c9ra10104g (PMC9048832; doi:10.1039/c9ra10104g)
Supplement: RA-010-C9RA10104G-s001 [file RA-010-C9RA10104G-s001.pdf]

Electronic Supplementary Material (ESI) for RSC Advances.
This journal is © The Royal Society of Chemistry 2020

Metal (Cd, Cr, Ni, Pb) removal from environmentally relevant waters using polyvinylpyrrolidone-coated magnetite nanoparticles

Jie Hong[a,b], Seyyedali Mirshahghassemi[b], Jamie Lead[b]*

[a]College of Environment, Zhejiang University of Technology, Hangzhou, Zhejiang, 310014, China

[b]Center for Environmental NanoScience and Risk, Department of Environmental Health Sciences Arnold School of Public Health, University of South Carolina, Columbia, SC, 29208, USA

* Corresponding Author: Jamie Lead, Email: jlead@mailbox.sc.edu;

Table S1. Correlation (R^2) obtained from pseudo-second-order equation of metal removal using PVP-Fe_3O_4 NPs.

	Cd	Cr	Ni	Pb
soft water	0.9994	0.9908	0.9896	0.9996
sea water	0.9753	0.9979	0.9974	0.9999

Table S2. Cd speciation and concentration (mol L^{-1}) in soft water and sea water by MINTEQ.

	Soft water	Sea Water
$Cd(OH)_2$ (aq)	1.845E-06	1.5055E-13
$Cd(OH)_3^-$	2.8891E-08	1.2408E-19
$Cd(OH)_4^{-2}$	7.8234E-11	1.7662E-26
$Cd(SO_4)_2^{-2}$	4.143E-08	1.4713E-07
Cd^{+2}	7.9342E-08	3.8722E-06
Cd_2OH^{+3}	3.8833E-13	4.4723E-14
$CdCl^+$	2.4415E-09	0.000045489
$CdCl_2$ (aq)	5.4408E-12	0.00003865
$CdOH^+$	3.3329E-07	6.6555E-10
$CdSO_4$ (aq)	7.9062E-08	8.0915E-07
$Cd(CO_3)_2^{-2}$	8.1764E-05	
$Cd(OH)_2$ (aq)	1.845E-06	
$CdCO_3$ (aq)	4.7928E-06	
$CdHCO_3^+$	1.5836E-09	

Table S3. Cr speciation and concentration (mol L^{-1}) in soft water and sea water by MINTEQ.

	Soft water	Sea Water
$Cr_2O_7^{-2}$	2.6774E-18	1.8322E-18
CrO_3Cl^-	8.1518E-24	3.6526E-21
$CrO_3SO_4^{-2}$	1.5748E-20	5.8925E-21
CrO_4^{-2}	1.7381E-05	0.000012363
H_2CrO_4 (aq)	8.3808E-22	7.2217E-22
$HCrO_4^-$	2.1219E-10	1.6741E-10
$KCr_2O_7^-$	5.0506E-21	2.4116E-20
$KCrO_4^-$	2.117E-08	1.0506E-07

Table S4. Ni speciation and concentration (mol L^{-1}) in soft water and sea water by MINTEQ.

	Soft water	Sea Water
$Ni(OH)_2$ (aq)	6.2793E-05	9.6897E-11
$Ni(OH)_3^-$	9.0213E-05	7.7252E-15
$Ni(SO_4)_2^{-2}$	1.8274E-10	1.1027E-08
Ni^{+2}	1.6743E-07	0.00013915
$NiCl^+$	2.0053E-11	6.3617E-06
$NiCl_2$ (aq)	3.718E-16	4.4968E-08
$NiOH^+$	1.0023E-06	3.5915E-08
$NiSO_4$ (aq)	1.4211E-07	0.000024726
$NiCO_3$ (aq)	1.6003E-05	
$NiHCO_3^+$	1.4438E-08	

Table S5. Pb speciation and concentration (mol L^{-1}) in soft water and sea water by MINTEQ.

	Soft water	Sea Water
$Pb(OH)_2$ (aq)	2.7798E-06	1.7576E-10
$Pb(OH)_3^-$	4.4331E-06	1.0759E-14
$Pb(SO_4)_2^{-2}$	3.6944E-11	1.9109E-07
Pb^{+2}	7.581E-11	5.3892E-06
Pb_2OH^{+3}	3.5365E-16	6.3022E-11
$Pb_3(OH)_4^{+2}$	1.2442E-11	4.1898E-15
$Pb4(OH)_4^{+4}$	8.0997E-18	2.707E-16
$PbCl^+$	8.8681E-13	0.000024073
$PbCl_2$ (aq)	1.0371E-15	0.000010736
$PbCl_3^-$	8.0245E-19	4.0799E-06
$PbCl_4^{-2}$	4.9306E-22	1.2295E-06
$PbOH^+$	1.0044E-07	2.131E-07
$PbSO_4$ (aq)	1.5781E-10	2.3527E-06
$PbCO_3$ (aq)	6.6368E-07	
$PbHCO_3^+$	3.8204E-11	
$Pb(CO_3)_2^{-2}$	4.0287E-05	

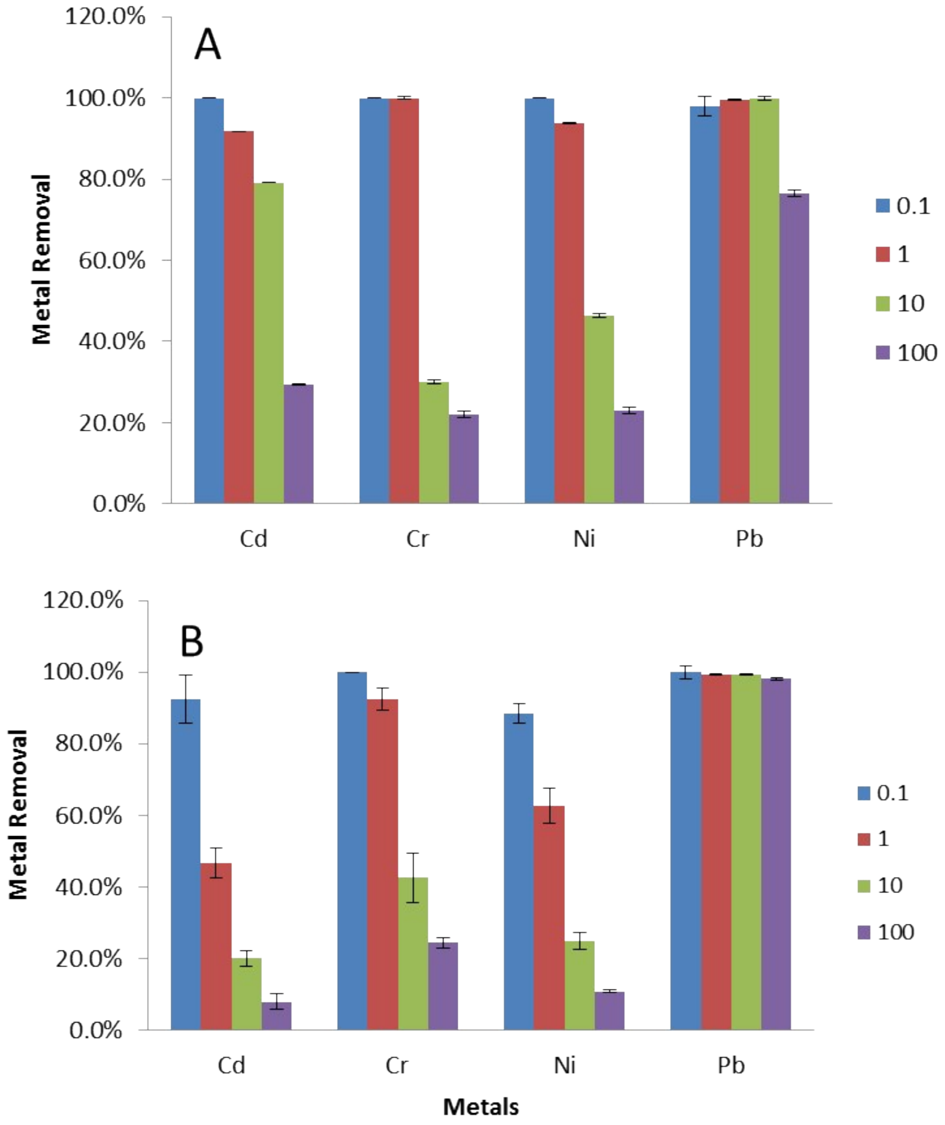

Figure S1. Effects of metal concentration (mg L^{-1}) on removal efficiency in different water media. (A) EPA soft water, (B) EPA sea water. Data are average of three replicates.
